# The IRB Reliance Exchange (IREx): A national web-based platform for operationalizing single IRB review

**DOI:** 10.1017/cts.2022.376

**Published:** 2022-03-23

**Authors:** Emily Sheffer Serdoz, Terri Edwards, Jill Pulley, Jenni Beadles, Julie Ozier, Paul Harris, Gordon R. Bernard, Todd W. Rice

**Affiliations:** 1 Vanderbilt Institute for Clinical and Translational Research, Vanderbilt University Medical Center, Nashville, TN, USA; 2 Human Research Protections Program, Vanderbilt University Medical Center, Nashville, TN, USA; 3 Department of Biomedical Informatics, Vanderbilt University Medical Center, Nashville, TN, USA; 4 Allergy, Pulmonary and Critical Care Medicine, Vanderbilt University, Nashville, TN, USA

**Keywords:** Single IRB review, single IRB platform, multicenter clinical research, sIRB, reliance, local considerations, sIRB coordination

## Abstract

For decades, the research community called for streamlined Institutional Review Board (IRB) review processes for multisite studies. Department of Health and Human Services and National Institutes of Health (NIH) recognized this need and implemented single IRB (sIRB) of record mandates. However, announcing mandates without sufficient operational guidance and tools is insufficient to foster the desired change. Nearly 4 years into implementation of the NIH’s sIRB mandate, operational challenges remain. Fortunately, NIH supports a web-based sIRB platform, the IRB Reliance Exchange (IREx), to facilitate sIRB communication and documentation. IREx has received continuous NIH funding supporting its evolution since 2011 and is now used by over 5,000 Human Research Protection Program and research personnel, 35 sIRBs, and 415 participating sites to operationalize sIRB review and approval on over 400 studies. IREx supports over 2300 reliance relationships with an average of 7 sites per study. The platform is continually used by sIRBs and relying sites, providing a valuable centralized portal for promoting a harmonized sIRB review process. IREx can promote transparency, standardize practice, minimize workflow variation, and mitigate the need for sIRBs to implement significant technical changes to their local electronic systems. IREx has proven to be nimble and adaptable with practice and policy changes over the past decade, as evidenced by continually increasing platform utilization.

## Background

Institutions, investigators, and funding agencies have long agreed that individual, local Institutional Review Board (IRB) review of multisite studies is inefficient. With increasing numbers of clinical trials occurring as multicenter trials, separate, independent review by each site’s IRB has been shown to be burdensome [1], inefficient [2, 3], and duplicative [4] and can result in significant variability and contradiction among IRB reviews and determinations [2, 4, 5]. Some findings also indicate that duplicative reviews may actually reduce the likelihood that studies meet traditional ethical standards for protecting human subjects [6, 7] and challenge the internal and external validity of trials [8]. Such problems are costly, require large portions of grant awards [9], may not provide adequate protection for human subjects, and can delay or prevent initiation of studies [1, 8]. The proposed solution was to encourage use of the single IRB (sIRB) of record for multisite studies.

In July 2011, the Office of Human Research Protections (OHRP) initiated the lengthy process of revising the regulations governing multisite IRB review in the USA by releasing an Advance Notice of Proposed Rulemaking (ANPRM). The ANPRM detailed revisions to the Common Rule that would facilitate “valuable research and reduc[e] burden, delay, and ambiguity for investigators” [10] and opened the way for public comment on the proposed changes. A subsequent Notice of Proposed Rule Making was released in 2015 [11] before a final, revised Common Rule, requiring the use of a sIRB of record for collaborative studies, was announced in January 2017 [12]. The final revised rule would then be amended twice in 2018 before becoming effective in January 2019. However, institutions were granted an additional 12 months before the sIRB requirement became effective in January 2020 [13]. Around the same time, the National Institutes of Health (NIH) mandated an sIRB for NIH-funded studies to “enhance and streamline the IRB review process for multisite research so that research can proceed as quickly as possible without compromising ethical principles and protections for human research participants” [14]. NIH followed a similar public comment and revision process that began with the announcement of an sIRB mandate in December 2014 [15], was finalized in June 2016 with an effective date of June 2017, and was ultimately postponed until January 2018 [16].

The fact that both sIRB mandates were delayed multiple times is testimony to the magnitude and complexity of this change for academic IRBs. The effective date extensions from both OHRP and NIH were intended to provide institutions time to align with and establish processes to support the mandates, but no centralized guidance was disseminated with the policies to inform how institutions might adjust their policies and electronic systems to comply. This paper discusses the operational gaps and opportunities created by the OHRP and NIH sIRB mandates and describes how an NIH-supported, web-based platform – the IRB Reliance Exchange (IREx) – has become a workflow solution for harmonizing the sIRB review process.

### Challenges Operationalizing the Single IRB Mandate

Nearly four years after full implementation of the NIH sIRB mandate, institutions and investigators now understand that an sIRB is required (with few exceptions) for multicenter studies. Establishing a common reliance agreement that avoided study-specific negotiations was anticipated to be one of the greatest hurdles to complying with the sIRB requirement [17]. Fortunately, the SMART IRB Agreement fulfilled this need [18] and has shown broad acceptability with more than 900 partner institutions [19]. However, creating processes and workflow-based tools for sIRB review remains a challenge. While the sIRB mandates and a common reliance agreement are necessary foundational elements of a national sIRB approach, they are not sufficient to efficiently comply with the mandates. “What is clear at this point is that simply specifying that an sIRB must provide the federally required ethics review for multisite trials, although an important step, will not by itself resolve the inefficiencies of regulatory review of multisite trials” [17]. Single IRBs, Human Research Protection Programs (HRPPs), coordinating centers and lead study teams, and relying site study teams need tools and resources to achieve the well-intended goals of the sIRB mandates. Four areas where tools could improve the current sIRB process are outlined below.

The first step to using an sIRB is establishing reliance. While the SMART IRB Agreement delineates most responsibilities of both the sIRB and relying sites, several “flexible” terms, such as indemnification and insurance, are not defined in the SMART IRB Agreement, but are instead outlined in a separate document called the SMART IRB Implementation Checklist and Documentation Tool (https://smartirb.org/assets/files/SMART_IRB_Agreement_Implementation_Checklist_FORM.pdf). This tool states that “[w]hile use of this tool is not required, Participating Institutions should document the selected options for each study in which they are involved. Both the Reviewing IRB and Relying Institutions should maintain a copy of the completed tool or alternative documentation for a study (e.g., a standard operating procedure) that is covered by the SMART IRB Agreement” [19]. Thus, while the SMART IRB Agreement affords flexibility in reliance relationships, the flexible terms must be determined “on a study-by-study or protocol-by-protocol basis” [20]. Given that responsibilities are likely to differ across studies and sIRBs, an electronic system is needed to facilitate communication and negotiations, streamline documentation, and provide a centralized, historical repository for all reliance relationships.

Once reliance is established for a study, perhaps the even greater challenge of collaborative institutional review begins. sIRBs must understand the local considerations of relying sites in order to review and approve them on a study, and relying sites need to communicate these considerations to the sIRB [21]. The SMART IRB Agreement and the NIH policy guidance [14] clearly state the relying site’s responsibility is to communicate to the sIRB “requirements of any applicable state or local laws, regulations, institutional policies, standards, or other local factors, including local ancillary reviews, relevant to the Research (‘Local Considerations’) ….” [19]. However, as Klitzman astutely points out, “[d]elineation of the type of information that sIRBs need to have, development of mechanisms to collect and transmit local knowledge effectively and efficiently, and clarification of the roles of such contextual information in single IRB reviews are critical” [20]. The federal mandates did not prescribe what questions sIRBs should ask or what information relying sites should provide for local considerations. Tools are needed to ensure adequate, but efficient communication, documentation, and review of local considerations from relying sites [20–23]. Rather than resorting to sIRB-specific questions and processes that change from one study to the next [21], sIRBs need help standardizing what is meant by “local considerations,” and relying sites would benefit from a systematic way of communicating this information to the sIRB rather than developing and learning new processes with each study.

In addition to tools that provide centralized repositories of documentation and standardize local considerations, workflow-based tools are needed to support the “sIRB coordination” required to keep relying sites moving through the sIRB review pipeline. The coordinating center or lead study team is typically responsible for sIRB coordination, which is described in the SMART IRB Overall Principal Investigator/Lead Study Team Guidance and Checklist (https://smartirb.org/assets/files/PI_checklist.pdf) [19] and includes educating relying site study teams about the sIRB process, ensuring all documentation is in place for each relying site, and submitting relying sites to the sIRB for review. The importance of sIRB coordination is evident in the NIH requirement to submit an sIRB plan with all multisite study applications [14] and has been noted by research networks who highlight the need for dedicated staff at the lead site to help manage the sIRB process and work with relying sites and the sIRB [24]. However, in addition, each relying site study team must follow their local IRB’s reliance process, which often involves a submission to the local IRB [25] in order for relying sites to document local considerations, before they can submit to the sIRB for review. This is a major shift from the traditional IRB review process of only a single submission to the local IRB. Significant communication and coordination are needed to ensure all sIRB stakeholders, including study teams, understand their roles, the order of operations, documentation requirements, as well as what and to where information needs to be submitted. Tools that can automate tracking of a relying site’s readiness for sIRB review, facilitate communication, and identify when and where bottlenecks arise are needed to support this critical sIRB coordination.

Finally, tools are needed to support systematic collection and evaluation of whether the sIRB mandates are streamlining the IRB review of multisite studies [26, 27]. Capturing efficiency and effectiveness metrics can help identify areas for process and policy improvement. The federal mandates will generate a large dataset from which to learn, and a centralized system to capture these data will ensure they are “accessible, collected, analyzed, and reported systematically” [27].

### A Web-Based Solution for Operationalizing the Single IRB Mandate

The IRB Reliance Exchange (https://IRBExchange.org) (IREx) is a web-based platform used by sIRBs, HRPPs, as well as lead and relying site study teams to support sIRB documentation and coordination. IREx has been in development by Vanderbilt University Medical Center (Vanderbilt) since 2011 with continuous NIH funding. IREx is currently supported by the National Center for Advancing Translational Sciences’ Trial Innovation Network (TIN), which has three sIRBs (Johns Hopkins School of Medicine, the University of Utah, and Vanderbilt (https://trialinnovationnetwork.org/elements/central-irb/)) supporting more than 70 TIN studies. The TIN sIRBs significantly informed the development of many IREx features early on and selected IREx as their common sIRB platform to harmonize processes across all TIN studies. The TIN now offers IREx as an sIRB resource for non-TIN sIRBs to manage federally funded, investigator-initiated, and industry-sponsored studies (https://www.irbexchange.org/p/studies/). In this section, we detail how IREx addresses the challenges summarized above, serving as a centralized, historical repository of reliance; facilitating documentation of local considerations; supporting sIRB coordination; and systematically capturing metrics from a broad array of sIRB studies.

IREx is a technology platform; it is not a reliance agreement. However, IREx can support reliance relationships based on the SMART IRB Agreement or any other IRB Authorization Agreement. Moreover, when the SMART IRB Agreement is used, IREx allows for rapid consensus building to operationalize the flexible terms of the SMART IRB Agreement using IREx’s Study-specific Reliance Plan (SSRP), which incorporates all elements of the SMART IRB Implementation Checklist and Documentation Tool. When agreeing to serve as the sIRB for a study in IREx, the sIRB indicates their preference for each flexible element. Relying sites can either accept or request changes to those preferences, which the sIRB can either accept or reject. Once the SSRP is accepted, a letter of reliance and a copy of the SSRP are automatically emailed to the sIRB and relying site with reference to the study. IREx makes the documentation process simple; communication is streamlined; and all information is available for historical reference. To date, over 2300 SSRPs have been documented. Relying sites typically accept the SSRP within 2 days of accessing a study in IREx, and, notably, 1072 SSRPs were accepted in 1 day or less.

The IREx platform continues to support relying sites as they move beyond indicating reliance for studies and begin documenting their local considerations. As the TIN sIRB system, IREx collaborated with the TIN sIRBs to develop comprehensive local considerations documentation, which is comprised of a high-level Institutional Profile (IP) and study-specific Human Research Protections (HRP) and Principal Investigator (PI) Surveys. While many elements of local considerations are protocol-specific (e.g., qualifications and credentials of key study personnel, conflicts of interest, institutional resources, ancillary reviews), the IREx IP captures relatively stable, institution-specific information that does not change on a study-by-study basis (e.g., Federalwide Assurance information, state-specific requirements such as required language for mandatory reporting to authorities, age of majority, circumstances affecting age of consent, record keeping requirements, specific language for subject injury, authorization of HIPAA waivers, and HIPAA authorization formatting requirements). Local information that is study-specific is documented by the relying site’s HRPP on the HRP Survey (note: while local ancillary reviews can be captured in the HRP Survey, these reviews are not required to be completed before the HRP Survey can be completed and the site submitted for sIRB review). Additionally, the PI Survey captures information about the conduct of the study and any procedures at a relying site that differ from the protocol. Thus, relying HRPPs and PIs collaboratively provide their local considerations in IREx, which ensures the HRPP is adequately aware of the study taking place at their institution and their institution’s involvement. Moreover, IREx allows iterative revisions to local documentation with corresponding communications to the lead study team or coordinating center and sIRB so the most up-to-date information is submitted for sIRB review. Since the release of the local considerations module in February 2018, 291 of 378 IREx studies (79%) have opted to capture local considerations in IREx rather than capturing this information via email or other methods. Additionally, 362 IREx institutions (87%) have completed their IP, which is required before a site can indicate reliance on an sIRB (fewer than 10% of IREx institutions have not used IREx to rely on an sIRB). Because the information on the IREx IP is broad and not specific to a study or protocol, IREx has made it publicly available on the IREx website as a resource for any institution using sIRB review, regardless of whether they are using the IREx platform (https://www.irbexchange.org/p/participants/).

IREx includes sIRB coordination features that the lead study team or coordinating center staff leverage to manage documentation. sIRB coordination features include granting site access to the study, tracking site progress and readiness for sIRB review, exporting local considerations for submission to the sIRB, and communicating site approvals from the sIRB. As stated in the SMART IRB Overall Principal Investigator/Lead Study Team Guidance and Checklist, “As part of preparing the IRB application, the Lead Study Team (or designee) must have a mechanism in place to obtain and collate information from Relying Site Study Teams and/or Relying Site Points of Contacts (POCs)…” [19] IREx fulfills this need so lead study teams do not have to create one-off tools. Moreover, IREx provides automated checklists to guide the lead study team through the sIRB process and dashboards to monitor site progress towards sIRB approval. IREx has over 400 lead study team members designated as “study managers” leveraging the sIRB coordination features.

**Fig. 1.**
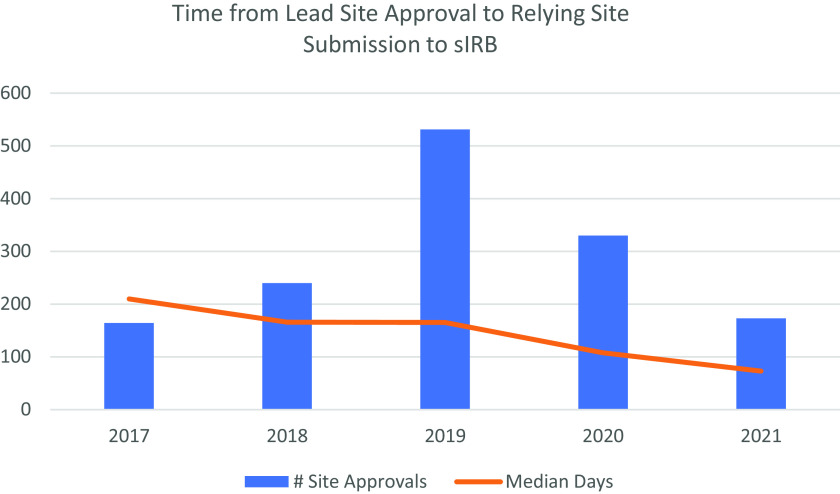
Days from lead site initial approval to relying site submission to the single IRB (sIRB), by year.

With the technology in place to standardize the sIRB process and support the sIRB process, IREx provides an opportunity to evaluate the efficiency of the sIRB mandate. Not only can IREx track the volume of reliances and studies using sIRB review, IREx also captures how long it takes relying sites to complete the sIRB review process – from submission and approval of the lead site through sIRB approval of relying sites. IREx data show that the median time for sIRB approval of the lead site is 33 days (*n* = 343), and relying sites receive initial sIRB approval within 12 median days of submission to the sIRB (*n* = 1718). While this reflects expeditious sIRB review of relying sites, it does not capture the full time to approval for relying sites. For example, before a site is submitted to the sIRB, the lead study team typically disseminates the sIRB-approved study materials to relying sites (e.g., protocol, consent templates); relying site study teams prepare their local submission; relying site HRPPs document local review; and the lead study teams prepare the relying site’s submission to the sIRB. In 2020, The IREx data available show the median time for these steps was 108 days (*n* = 330). Notably, relying site HRPPs documented local reviews in a median of 17 days, and lead study teams required a median of 16 days to prepare a site submission. While delineating the review parameters of the sIRB and relying site HRPP have been challenging and can cause delays, our data indicate other factors – likely relying site study teams navigating their local reliance procedures and preparing their local submission – may cause more significant delays. However, the process is improving (see Fig. 1). A centralized portal, like IREx, that is used by multiple sIRBs and relying sites is illuminating these critical impediments and can inform future policies, procedures, IREx enhancements, and best practices.

IREx is a useful tool today and is continuously evolving. When initially launched in October 2016, IREx could capture study-specific documentation of reliance, lead site approvals, relying institution approvals, and communications (email notifications) of these events. Five years later, IREx is a customizable platform offering features that sIRBs can turn on or off on a study-specific basis, such as whether to require indemnification, offer non-SMART IRB Agreements, and identify study-specific points of contact. IREx captures dynamic information from relying sites and triggers notifications based on actions needed or completed. Table 1 outlines the development of major IREx features over the past 5 years.

**Table 1.** Major IRB Reliance Exchange (IREx) feature releases

Major system enhancements	Feature description	Release date
IREx Launch	Single IRBs (sIRBs) and relying sites have a system to: (1) document reliance and (2) disseminated sIRB approvals to relying sites on a study-by-study basis.	October 2016
Accommodate Federalwide Assurance (FWA) component sites	Relying sites can include component sites in the system to further delineate where research is occurring and/or indicate multiple study teams are participating on a study	February 2017
Facilitate HRPP staff that oversee multiple FWAs (“Multisite Liaisons”)	Human Research Protection Program (HRPP) leaders may oversee multiple FWAs for their organization so IREx offers different user interfaces to streamline documentation and usability for these users	May 2017
Facilitate Lead Study Teams/Coordinating Center Functions (“Study Manager”)	Coordinating center staff and lead study team members can manage site access to IREx, monitor site progress completing the required documentation that occurs prior to sIRB review, and disseminate sIRB approvals to sites.	July 2017
Capture local considerations	sIRBs have the option to capture study-specific local considerations from relying sites in IREx. This includes the Institutional Profile and Human Research Protections Survey completed by the HRPP, as well as a Principal Investigator (PI) Survey.	February 2018
Align SSRP with SMART IRB Implementation Checklist and Documentation Toolkit	IREx aligned the study-specific reliance plan (SSRP) with the SMART IRB tool that outlines the flexible elements of reliance.	April 2018
IRB Dashboards for cross-study progress tracking	sIRBs and relying site dashboards provide an overview of each study, as well as outstanding actions.	August 2018
Institutional Profiles (IP) publicly available	IREx IPs are made publicly available on https://IRBExchange.org for HRPPs to reference when considering whether to serve as the sIRB for a site or to provide when relying on an sIRB	October 2018
Track completion of Indemnification	sIRBs that require terms of indemnification in addition to the SMART IRB Agreement can now track whether sites have completed the requirement. Relying sites are unable to indicate reliance until the sIRB’s indemnification terms are executed.	February 2019
Distinguish sIRB and Lead Site, when needed	For some studies, the sIRB institution is not the institution of the lead PI. IREx can distinguish the sIRB from the lead site and ensure the required documentation is in place to support reliance between the lead investigator’s institution and the sIRB.	November 2019
Provide Application Program Interface (API) for sIRBs	IREx API supports the bi-directional exchange of data between a sIRB’s electronic IRB (e-IRB) system and IREx. The IREx API facilitates the process of: (1) creating a study based on information from the sIRB’s e-IRB system; (2) posting initial approval for the lead site from the sIRB’s e-IRB system; (3) exporting site-specific local considerations to the sIRB’s e-IRB system; and (4) posting initial approval for relying sites from the sIRB’s e-IRB system.	February 2020
Document PIs engaging multiple FWAs on study (Combo sites)	When a single PI engages multiple FWAs on a single study, reliance and local considerations documentation are required from each FWA. IREx now captures this information from each FWA appropriately.	May 2020, Enhanced November 2021
Track use of non-SMART IRB Agreements	Some sites are unable to join the SMART IRB Agreement so IREx now allows sIRBs to offer one-off reliance agreements to relying sites, while still using IREx to document reliance and local considerations.	March 2021
Provide data exports for sIRBs and relying sites	sIRBs and relying sites can export data for all their studies in IREx. The sIRB export provides aggregate information like the # of sites; # sites with local considerations complete; and # of sites with initial sIRB approval. The sIRB can also export detailed information about the time required for relying sites to document reliance and local considerations, as well as time for each approval for relying sites. Relying sites can export the dates of each reliance on a sIRB and the dates of all sIRB approvals (initial, continuing review, amendments).	May 2021
Document Study and Site Closures	IREx can capture documentation when studies and/or sites are closed, including the date of and reason for the closure, as well as any relevant documentation.	September 2021
Track reliance for sites that are engaged in research, but not enrolling	When sites are engaged in research, but are not enrolling participants, they must document reliance, but they do not have the same types of approval documents as engaged sites (e.g., consent forms). IREx has added a label to distinguish sites that are not enrolling or have closed to enrollment.	December 2022
Ability to add custom document types and descriptions	The sIRB and Study Manager can now customize/relabel document types for “Additional IRB Approval Documents” and “Others.” These document types will no longer have the word “Other” as a prefix in the title.	January 2022
Streamlined access to approval documents for relying sites	Relying sites can now access all approval documents for their site on a single tab and download all documents – whether global documents for the entire study or site-specific – in a single click. Additionally, new descriptive information can be provided to help distinguish study amendments from one another.	March 2022

### Increasing Use of IREx

**Fig. 2.**
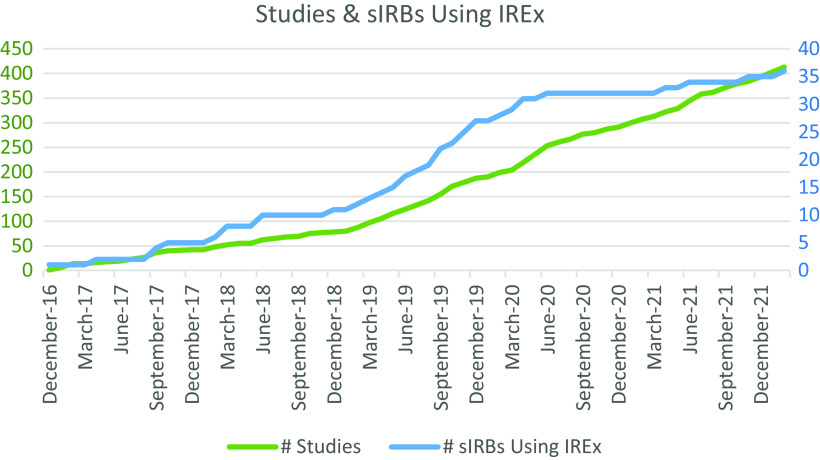
Studies and single IRBs (sIRB) using IREx, by year.

Since launching as the TIN sIRB system in December 2016, IREx has amassed 415 partner institutions and 409 studies, more than 300 of which come from outside the TIN, establishing IREx as a national resource for any sIRB. To date, the IREx Support Team has trained over 120 sIRB staff and more than 200 lead study team members or coordinating center staff. More than 35 academic sIRBs in 19 states use IREx to support one or more of their sIRB studies, and 70% of these sIRBs use IREx for multiple studies. IREx continues to be leveraged by new sIRBs every year, growing by more than 50% each year prior to COVID in 2020 (see Fig. 2). We know of no other electronic platform that is so extensively used to support the full sIRB review and approval process, not just documentation of reliance. sIRBs using IREx average 7.2 relying sites per study, and relying sites use IREx for an average of 5.4 studies (minimum = 1; median = 2; max = 55). Such broad use of a common platform greatly supports standardization in process that can result in efficiencies.

### Challenges to Increasing sIRB Portal Utilization

The IREx platform has a long history of evolving and adapting to support changes in policy and best practices. One limitation is that development is often reactive – based on the announcement of new policies and practices. As a result, IREx platform solutions may be developed concurrently to local institutional solutions or process changes. When this happens, institutions are less likely to modify their process again to leverage the IREx solution because they already have a working solution in place for their institution.

A second challenge that arises when building a platform for a nascent sIRB workflow is simply keeping up with demand. While IREx is responsive and continuously growing (see Fig. 2), we know of additional workflow gaps in need of a technical solution. For example, relying site study teams are frequently confused about when their site is formally approved and ready for enrollment. Anecdotally, we have heard of relying site investigators either fully circumventing their local HRPP and submitting to the sIRB for review without any input from their HRPP or beginning the study after their HRPP documented local considerations, entirely unaware that they needed sIRB review and approval. Because this is a communication issue and the process and requirements vary from IRB to IRB, IREx could provide a central hub for each party to ensure all the necessary steps are complete.

A final challenge is that IREx is not a full-fledged electronic IRB (e-IRB) system. IREx is a separate, cloud-based Software as a Service (SaaS) application that is used in combination with the local e-IRB system. When the sIRB mandates were announced, many institutional e-IRB systems did not have built-in mechanisms to support the sIRB workflow and documentation, such as submissions from relying sites. This created a need for either development and expansion of e-IRB systems, or the use of a supplement, external system like IREx. Modifying e-IRB systems takes significant time and can be costly, and, while use of an external system like IREx is cost-free, it requires workflow adjustments as well as education and user training for IRB staff. Both paths have challenges; however, the IREx platform offers an Application Programming Interface to communicate and exchange information with e-IRB systems and mitigate the need for significant workflow adjustments and staff training on a new system. Moreover, use of a common, standalone system across sIRBs promotes standardization of process rather than requiring relying sites’ HRPPs and/or study teams to navigate idiosyncratic e-IRB systems on a study-by-study basis.

## Discussion

The academic research community’s pleas for changes to the IRB review of multisite studies have been answered from a policy perspective. The USA has a national sIRB mandate and a national reliance agreement (SMART IRB). However, institutions and study teams have not had the technical tools to implement the sIRB mandates. In order to truly “eliminate unnecessary burdens that delay research and frustrate researchers and participants alike” [28], the research community needs technical infrastructure to streamline sIRB documentation, facilitate standard operating procedures, generate efficiencies, and support evidence-based changes to existing policies and processes. IREx has a decade-long history of growth, evolution, and utilization, despite any requirements to use it. As a common sIRB platform, IREx can provide stability and standardization to the sIRB process, which in turn could result in great efficiency for sIRB review of multisite studies.
